# Low human beta-defensin-2 levels in the sputum of COPD patients are associated with the risk of exacerbations

**DOI:** 10.1186/s12890-023-02364-0

**Published:** 2023-03-31

**Authors:** Shengchuan Feng, Yuqiong Yang, Fengyan Wang, Weijuan Shi, Jiaxuan Xu, Guoyan Tang, Jiaxing Xie, Nanshan Zhong, Zhenyu Liang, Rongchang Chen

**Affiliations:** 1 Guangzhou Institute of Respiratory Health, State Key Laboratory of Respiratory Disease, National Clinical Research Center for Respiratory Disease, National Center for Respiratory Medicine, The First Affiliated Hospital of Guangzhou Medical University, 510120 Guangzhou, China; 2 Department of Pulmonary and Critical Care Medicine, Shenzhen Institute of Respiratory Diseases, Shenzhen People’s Hospital (The Second Clinical Medical College, Jinan University; The First Affiliated Hospital, Southern University of Science and Technology), 518020 Shenzhen, China

**Keywords:** Chronic obstructive pulmonary disease (COPD), hBD-2, Exacerbations, Microbial colonization, Cytokines

## Abstract

**Rationale:**

Chronic obstructive pulmonary disease (COPD) is a complicated chronic inflammatory disease. It is important to investigate the characteristics of acute exacerbation of COPD to develop new therapeutic strategies.

**Objective:**

This study aimed to determine the relationship between the human beta-defensin-2 (hBD-2) levels and aggravation of COPD.

**Methods:**

We detected the sputum hBD-2 level of 254 patients from Guangzhou, China, for 2 years. The study participants were categorized into the COPD group (n = 203, GOLD 0–4) and the control group (n = 51, 40–79 years old). At baseline, 12th month, and 24th month, we detected the sputum hBD-2 level and levels of cytokines, such as CXCL10, CXCL11, and IFN.

**Results:**

At baseline, there were no significant differences in the sputum and serum hBD-2 levels between the patients and the controls. However, the sputum hBD-2 levels of patients who had at least one symptom aggravation over the next 2 years were significantly lower than those of patients without any exacerbations (1130.9 ± 858.4 pg/mL vs. 2103.7 ± 1294.2 pg/mL, respectively; p = 0.001). Nevertheless, there were no statistically significant differences in the sputum hBD-2 levels between patients (no aggravation history) and controls (2084.9 ± 1317.6 pg/mL vs. 2152.5 ± 1251.6 pg/mL, respectively; p = 0.626). We used a logistic regression model to assess the relationship between aggravation and sputum hBD-2 levels. Interestingly, we found that low hBD-2 level (< 1000 pg/mL) was significantly associated with exacerbations. Specifically, patients with low hBD-2 levels were more likely to experience exacerbations in the next 12 months (0.333 vs. 0.117; p = 0.001). Moreover, we compared the hBD-2 levels between controls and patients with GOLD 3–4 and found that participants with bacteria (+) and/or viruses (+) had an association between hBD-2 level and disease severity (p = 0.02).

**Conclusion:**

Patients at risk of exacerbations are more likely to have lower sputum hBD-2 levels. These results have important implications for future therapies for COPD.

**Supplementary Information:**

The online version contains supplementary material available at 10.1186/s12890-023-02364-0.

## Introduction

Human beta-defensin-2 (hBD-2), an anti-microbial peptide, promotes host defense by destroying the cell membranes of microorganisms. It cannot induce the migration of memory T cells and dendritic cells [1–3]. It is one of the components of innate and adaptive immunity [4, 5].

Normally, a large number of different bacteria colonize the upper respiratory tract of humans, such as *Haemophilus influenzae, Streptococcus pneumoniae*, and *Mycobacterium catarrhalis*, which are the normal microflora of the oropharynx. Potential pathogenic microorganisms (PPMs) refer to bacteria that have colonized the lower respiratory tract and mainly include *H. influenzae, Pseudomonas aeruginosa*, and *Enterobacter*. PPMs can cause inflammation and have a significant correlation with the exacerbation frequency and severity of COPD [6–8].

Moreover, respiratory viral infection, especially rhinovirus, is one of the causes of acute exacerbations of COPD. In rhinovirus infections, subsequent bacterial infection is detected in 60% of COPD patients. Bacterial and viral infections are important causes of acute exacerbations of COPD and may lead to a progressive decline in lung function and increased mortality [9–13].

The levels of cytokines, such as TNF-α, IL-1, IL-6, and IL-17 A, are increased in COPD patients. Similarly, the counts of neutrophils, macrophages, and T cells in the sputum or blood alcohol level (BAL) of COPD patients are also increased. The precise pathological roles of these cytokines and immune cells are uncertain. Their increased levels may be related to the poor efficacy of treatments that target TNF-α [14, 15] or p38 [16, 17]. Our current understanding of the pathogenesis of COPD is limited. Although COPD is considered an inflammatory condition, several anti-inflammatory treatments have failed to show positive effects in COPD.

Acquired immunodeficiency may play a role in the progression of COPD [18, 19]. Furthermore, the immune response to vaccinations may be impaired in patients [20]. Moreover, evidence also suggests that the mechanisms underlying innate immunity, such as the phagocytic activity of alveolar macrophages, are impaired [21]. Finally, some studies showed decreased levels of anti-microbial peptides in the upper airways of COPD patients [11, 22].

We hypothesized that the levels of hBD-2 and cytokines (such as TNF-α, IL-1, IL-6, and IL-17 A) are significantly different between controls and patients, and may predict the acute exacerbations of COPD. We aimed to improve our understanding of the relationship between sputum hBD-2 level and acute exacerbations of COPD [23].

## Participants and methods

### Study design

We performed a 2-year, single-center, observational study in Guangzhou, China. We enrolled 203 COPD patients (GOLD 0–4) and controls (51 healthy individuals). All participants were aged 40–79 years (Table 1). In addition to the enrolment visit (visit 1), three other follow-up visits were planned: visit 2 (baseline), visit 3 (1-year assessment), and visit 4 (2-year assessment). Between visits 2 and 4, the participants were assessed using symptom questionnaires; medical history; vital signs; chest X-ray; blood tests for hematology, biochemistry, and biomarker analysis; and lung function tests. In cases of COPD exacerbation, an extra clinic visit (visits 11.1–11.3) was performed within 48 h. More details about the study protocol are available in a previously published article [23].

**Table 1:** Baseline patient demographics (mean ± SD).

Variable	Patients n = 203	Controls n = 51	P Value
Female/Male (male %)	36/167 (82)	30/21 (41)	< 0.05
Age at Baseline	64.7 ± 7.8	59.5 ± 7.5	< 0.05
Number of Pack Years	28.7 ± 26.0	6.1 ± 13.6	< 0.05
Current smoker (%)	67 (33)	7 (14)	< 0.05
Former smoker (%)	84 (41)	10 (20)	< 0.05
Never smoker (%)	52 (26)	34 (66)	< 0.05
BMI (kg/m2) Derived	22.9 ± 3.2	24.0 ± 3.2	
FEV1 (L)	1.78 ± 0.70	2.28 ± 0.57	< 0.05
FEV 1 Post (L)	1.90 ± 0.70	2.28 ± 0.60	< 0.05
FEV1/FVC (%)	61.6 ± 16.2	79.9 ± 5.0	< 0.05
FRC (L)	3.96 ± 1.38	3.03 ± 0.75	< 0.05
FVC (L)	3.00 ± 0.78	3.00 ± 0.80	
FVC Post (L)	3.06 ± 0.74	3.00 ± 0.78	
IC (L)	2.19 ± 0.58	2.24 ± 0.56	
RV (L)	3.25 ± 1.68	2.23 ± 0.50	< 0.05
TLC (L)	6.14 ± 1.39	5.25 ± 1.06	< 0.05
DLCO (mmol/ kPa x min)	19.26 ± 5.61	22.86 ± 4.46	< 0.05

### Study population

We recruited COPD patients (n = 203, GOLD 0–4) and lung-healthy controls (n = 51). All participants were aged 40–79 years. The patients were stratified into four groups (GOLD 0, GOLD 1, GOLD 2, and GOLD 3–4), according to the disease severity. GOLD 0 was defined as chronic cough (without any other condition explaining the cough) and expectoration for at least 3 months in each of the two consecutive years, a postbronchodilator FEV1/FVC ≥ 0.7, and FEV1 ≥ 80% predicted normal (PN). GOLD 1, 2, and 3–4 were characterized by FEV1 ≥ 80% PN, FEV1 50–79% PN, and FEV1 < 50% PN, respectively.

### Inclusion and exclusion criteria

#### Inclusion criteria

The following inclusion criteria were used for participants with COPD (GOLD 0–4):

(1) Guangzhou residents; (2) aged 40–79 years; (3) fulfilling one of the following conditions: (1) a diagnosis of COPD (GOLD 1–4) according to GOLD 2008 or (2) at risk for COPD (GOLD 0); and 4. able to read and understand the local language so they could complete the study questionnaires.

The following inclusion criteria were used for participants in the control group:

(1) Guangzhou residents; (2) postbronchodilator FEV1/FVC ≥ 0.7 and FEV1 ≥ 80% PN without any respiratory diseases or symptoms; (3) age- and sex-matched males and females (aged 40–79 years); and (4) able to read and understand the local language so that they could complete the study questionnaires.

#### Exclusion criteria

The following exclusion criteria were used for COPD patients (GOLD 0–4):

(1) Asthma (e.g., as defined by GINA 2008), without symptoms or diagnosis of COPD; (2) major diseases or disorders, such as cardiovascular disease, lung disease other than COPD, digestive tract diseases, liver diseases, kidney diseases, neurological diseases, musculoskeletal diseases, endocrine disorders, metabolic diseases, malignant tumors, and major physical injuries; (3) chronic respiratory failure, including the need for regular oxygen therapy; (4) severe acute exacerbation of COPD within the last 1 month before enrollment (referring to the use of antibiotics) and/or oral/systemic glucocorticoids or hospitalization due to COPD exacerbation); (5) participated in another study within the 3 months before enrollment, used study drug, or donated blood (> 500 mL); (6) had an inpatient plan during the study period; and (7) change in COPD maintenance medication within the last 1 month before enrollment, or start a new COPD medication.

The following exclusion criteria were used for participants in the control group.

(1) Major diseases or disorders, such as cardiovascular diseases, lung diseases other than COPD, gastrointestinal diseases, liver diseases, kidney diseases, neurological diseases, musculoskeletal diseases, endocrine diseases, metabolic diseases, malignant tumors, and major physical injuries; (2) any chronic respiratory symptoms or chronic respiratory symptoms illness; (3) participated in another study within the last 3 months before enrollment or used study medication or donated blood (> 500 mL); and (4) hospitalization planned during the study period.

### Measurements

Pulmonary function measurements were performed according to the American Thoracic Society (ATS)/European Respiratory Society (ERS) criteria [24]. Total lung (TLC), functional residual capacity (FRC), deep inspiratory capacity (IC), and residual air volume (RV) were measured using the plethysmographic or nitrogen washout technique. The diffusing capacity of carbon monoxide (DL_CO_) was determined using the single-breath method.

Biomass and categories of viruses and bacteria in sputum samples and nasal and throat swabs were measured using polymerase chain reaction (PCR) or sputum cultures (for more details, see Additional file 1: Tables S1 and S2). The levels of hBD-2 and various cytokines (such as CXCL10, CXCL11, and IFN) in the sputum and serum were measured by ELISA (Phoenix Pharmaceuticals) and V-plex assay (Mesoscale Discovery).

### Statistical analysis

All statistical analyses were performed using R software (version 4.2.0). The ggplot2 package (version 4.2.0) was used to draw the figures. We calculated the mean ± standard deviation (SD) or interquartile range for the levels of hBD-2 and cytokines (CXCL10, CXCL11, and IFN). We used the Wilcoxon rank sum test to assess the differences between the two groups. The Wilcoxon signed-rank sum test was used to analyze paired data without a normal data distribution. The Kruskal-Wallis test was used to analyze the differences in data without a normal data distribution. The association between two categorical variables was evaluated using the chi-square test. For all statistical tests, p < 0.05 was considered statistically significant.

For correlation network analysis, we used the psych package (version 4.2.0) to calculate the correlation coefficients (method: Spearman) and p values. P < 0.005 was regarded as the threshold for filtering data. We used Cytoscape (version 3.9.1) to perform the network analysis [25, 26]. Connectivity (stress) was calculated using Centiscape 2.2 in Cytoscape. The edge between the two points in the graph indicates that the correlation coefficient of the two cytokines had a statistically significant difference (p < 0.005). The correlation coefficient for the cytokines was weighted and shown in the graph as the width of the edge. In the diagram, the colors of the virus-related cytokines (CXCL10serum, CXCL11sputum, and IFNγsputum) were red or light green. The degree of connectivity is related to the area of dot. Typically, the larger the area, the higher the degree of connectivity.

## Results

### Changes in sputum hBD-2 levels in healthy controls and patients

**Fig. 1:**
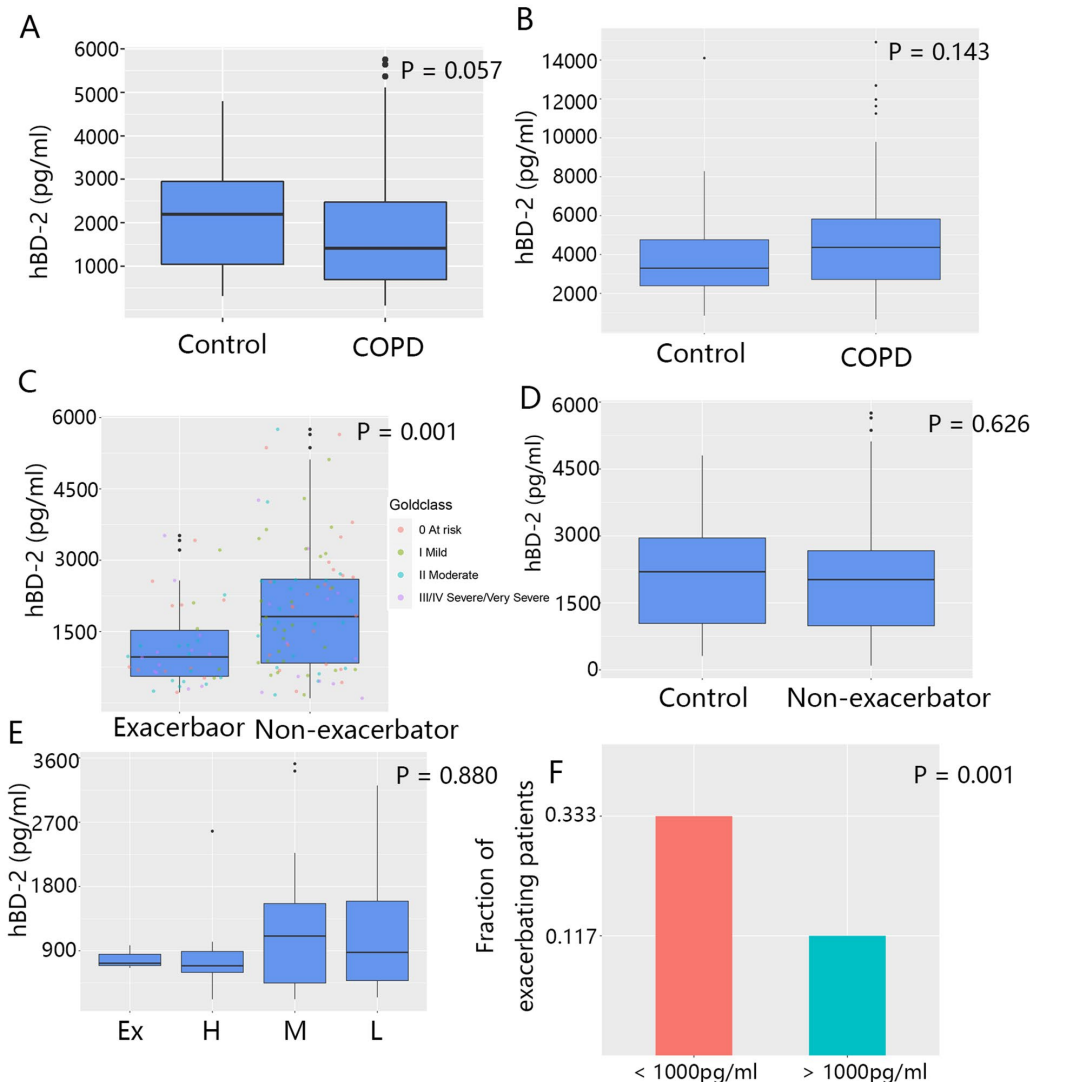
Sputum and serum hBD-2 levels in COPD exacerbators, non-exacerbators, and controls at baseline. (A–B) hBD-2 levels in the sputum (A) and serum (B) in patients and controls measured by ELISA. (C) The hBD-2 levels of patients who never experienced exacerbation were compared with those of patients who experienced exacerbation at least once. (D) The sputum hBD-2 levels of patients without any exacerbation were compared to those of controls. (E) Patients were categorized into four subgroups according to the frequency of exacerbations. The sputum hBD-2 levels were compared between subgroups (ex-high, > 2 per year; high, 1.5–2 per year; medium, 1–1.5 per year; and low, < 1 per year). (F) According to a cutoff level (1000 pg/mL), patients were grouped into two subsets: the higher subset (> 1000 pg/mL) and the lower subset (< 1000 pg/mL). The proportion of patients experiencing an exacerbation within the following 12 months was calculated. Statistical methods: Kruskal-Wallis (A, B, D, and E); Wilcoxon (C); and chi-square test (F) (for more details about Fig. 1, see Additional file 1: Table S5)

At baseline, compared to the control group, COPD patients had no significant difference in terms of the sputum hBD-2 levels. However, compared to the former, the latter group showed a trend toward lower hBD-2 levels (2152.5 ± 1251.6 vs. 1716.9 ± 1248.6 pg/mL, respectively; p = 0.057; Fig. 1A). Additionally, the serum hBD-2 level was not different between the two groups (3899.1 ± 2561.3 vs. 5204.4 ± 3704.7 pg/mL; p = 0.143; Fig. 1B).

Furthermore, at baseline, patients without any exacerbation during the study had a significantly higher sputum hBD-2 level than patients who experienced more than one exacerbation (2103.7 ± 1294.2 vs. 1130.9 ± 858.4 pg/mL, respectively; p = 0.001; Fig. 1C). Moreover, the control group and patients without any exacerbation during the study had no significant difference in the sputum hBD-2 level (2152.5 ± 1251.6 vs. 2084.9 ± 1317.6 pg/mL, respectively; p = 0.626; Fig. 1D).

Patients were categorized into four subgroups (ex-high, high, medium, and low), according to the frequency of exacerbations (see Fig. 1 for more details). We hypothesized that the sputum hBD-2 level is negatively correlated with the frequency of exacerbations. However, we did not find any difference in the sputum hBD-2 level among the subgroups (p = 0.880; Fig. 1E).

In addition, at baseline, we grouped patients into two subsets according to a cutoff of the median level of sputum hBD-2 (1000 pg/mL). Then, we used logistic regression to investigate the relationship between exacerbations and other variables. The results showed that exacerbation was not associated with age, gender, or smoking status (p = 0.540, p = 0.131, and p = 0.562, respectively) but was associated with the hBD-2 level (p = 0.002). Additionally, we found that patients who had a lower hBD-2 level were more likely to experience exacerbations in the next 12 months (0.519 vs. 0.204, respectively; p < 0.005; Fig. 1F).

### Variation in pathogens at baseline and exacerbations

**Fig. 2:**
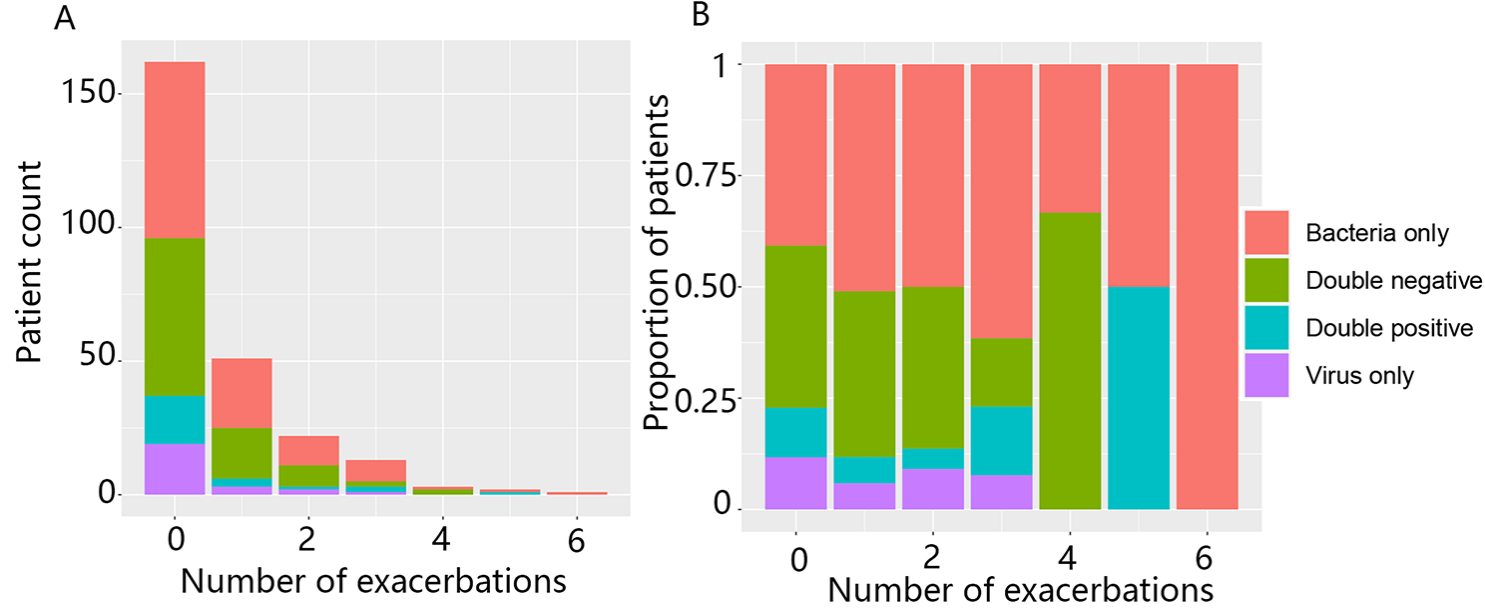
Association of pathogen colonization status at baseline (Only Bact+, Only Vir+, Both+, and Both-) with the frequency of exacerbations in patients. Patients were grouped according to the times of exacerbations during the 2-year study, and the number (A) or proportion (B) of patients who were positive or negative for bacteria or viruses was calculated

In patients with stable disease (visits 2–4), viruses (+) and bacteria (+) accounted for 31% and 38% of the pathogens, respectively. However, at the time of exacerbations (visits 11.1–11.3), viruses (+) and bacteria (+) accounted for an increased proportion than at baseline (i.e., 52% and 41%, respectively) (Additional file 1: Table S3). Furthermore, bacteria (+) or viruses (+) did not show significant differences between COPD patients and controls (Additional file 1: Table S4). Moreover, during the study, patients with both bacteria (+) and viruses (+) accounted for 11–15% of all patients (Additional file 1: Table S5). Patients with bacteria (+) and/or viruses (+) at baseline (visit 2) did not have an increased risk of exacerbation (Fig. 2).

**Fig. 3:**
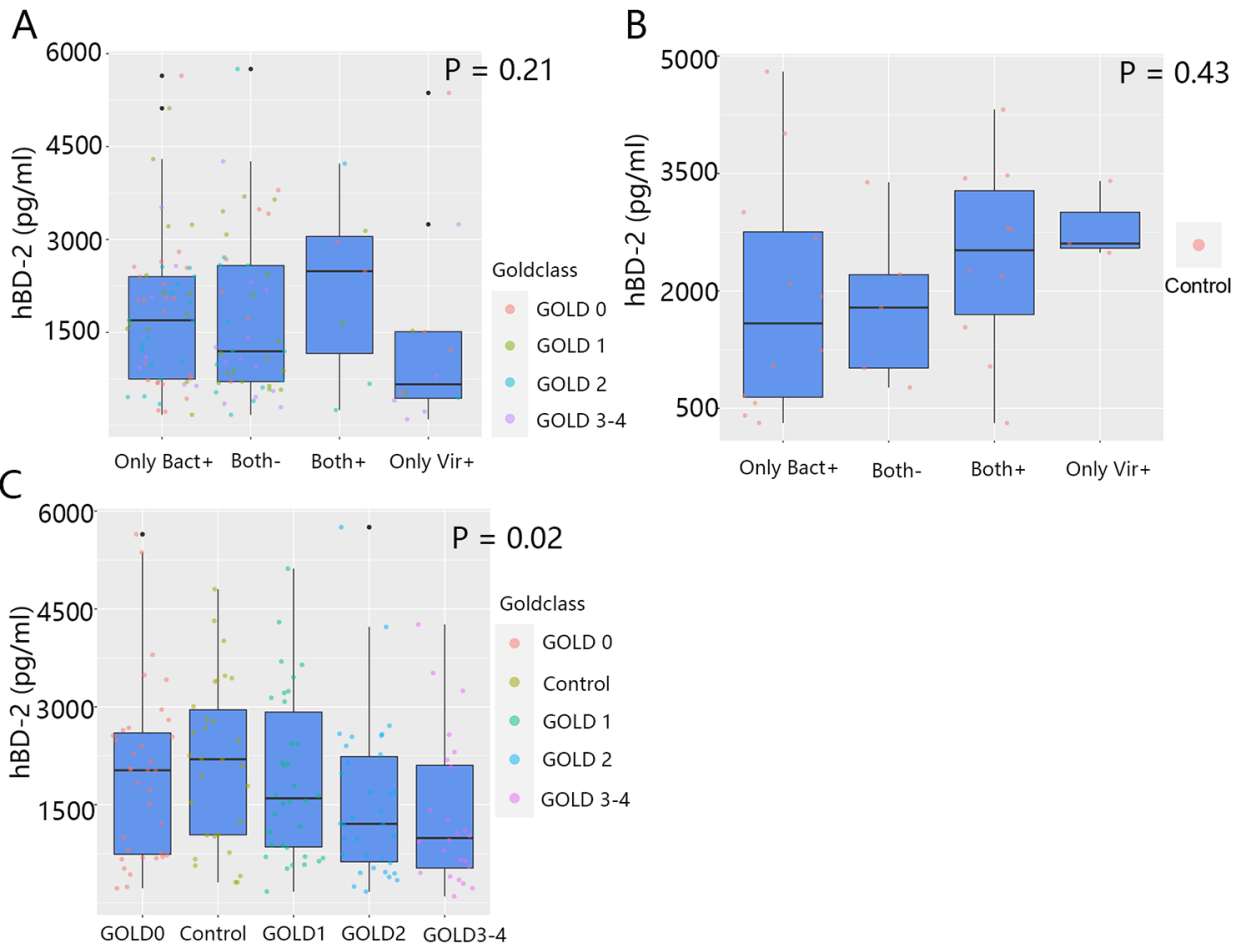
At baseline, the sputum hBD-2 levels were subdivided by colonization status (Only Bact +, Only Vir +, Both +, Both-) and disease severity (GOLD 0, 1, 2, and 3–4). The sputum hBD-2 levels of COPD patients (A) and controls (B) were compared according to the colonization status. (C) The sputum hBD-2 levels in controls and COPD patients (Bact + or/and Vir +) were compared according to the disease severity. Statistical methods: Kruskal-Wallis test was used to detect significance (for more details about Fig. 3, see Additional file 1: Table S5)

At baseline, the sputum hBD-2 level was not significantly associated with pathogen colonization in patients and controls (p = 0.21 and p = 0.43, respectively; Fig. 3A and B). Although the percentage of colonization did not correlate with the GOLD classification (Additional file 1: Table S4), we found that the hBD-2 levels were negatively correlated with the GOLD classification. The hBD-2 level in controls was the highest, whereas the hBD-2 level in patients (GOLD 3–4) was the lowest (control group, 2216.34 ± 1298.22; GOLD 0, 1826.30 ± 1353.02; GOLD 1, 2165.52 ± 1303.80; GOLD 2, 1518.09 ± 999.67; GOLD 3–4, 1355.53 ± 1190.34 pg/mL; p = 0.02; Fig. 3C).

**Fig. 4:**
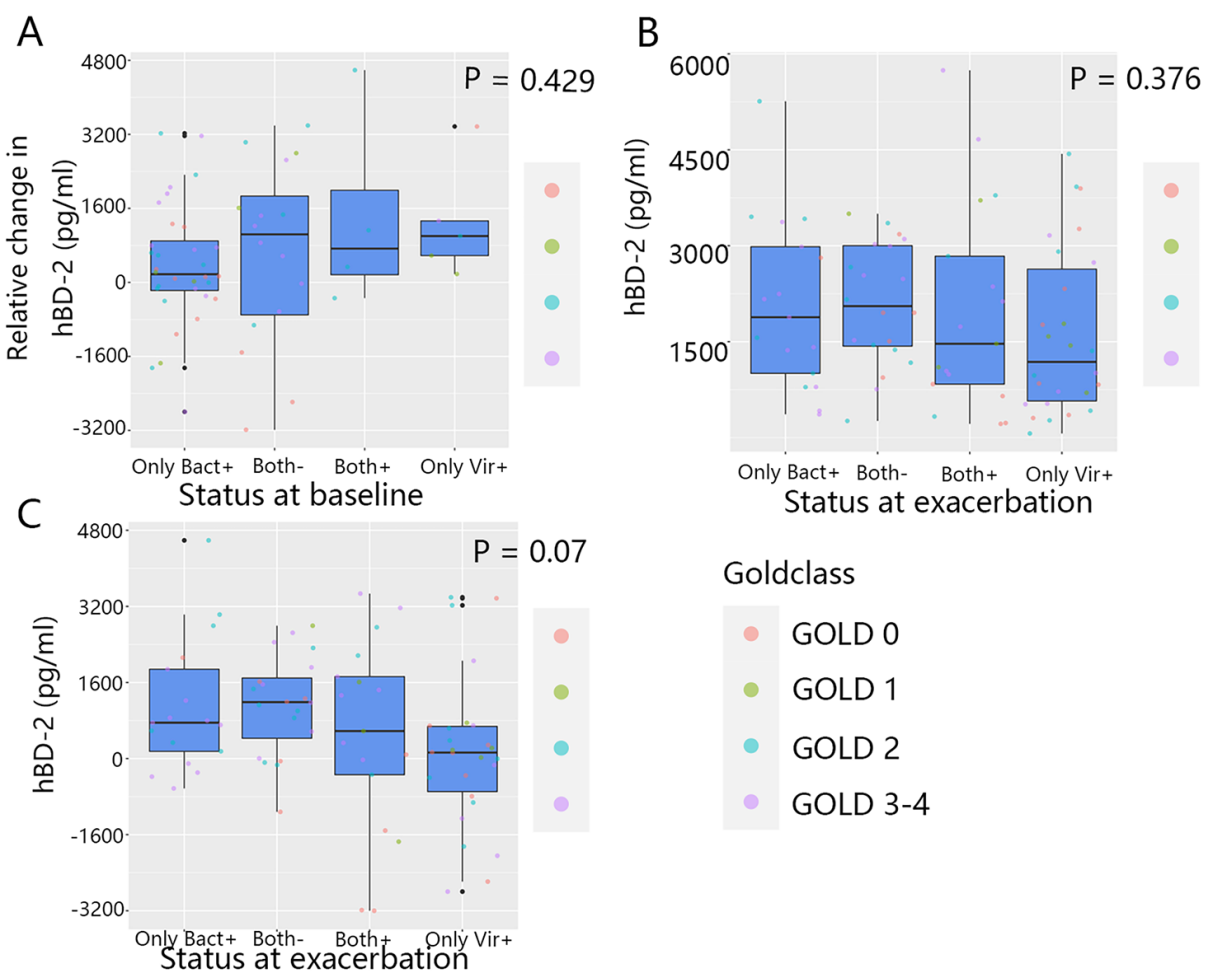
Relationship between sputum hBD-2 levels and the pathogen colonization status (Only Bact+, Only Vir+, Both+, Both-) in exacerbations. (A) The relative change in sputum hBD-2 level from baseline to exacerbations was calculated as per status. The relative change in sputum hBD-2 level was compared among patients according to the status at baseline. (B, C) The sputum hBD-2 levels were compared at the time of exacerbation, and (B) relative change in sputum hBD-2 level (C) was compared in patients according to the colonization status at the time of exacerbation. Statistical methods: Kruskal-Wallis test was used to determine significance (for more details about Fig. 4, see Additional file 1: Table S6)

At the time of exacerbations (visits 11.1–11.3), the sputum hBD-2 level was increased in the majority of patients, which did not depend on the presence of bacteria or viruses in the airways at baseline (only bacteria (+): 371.48 ± 1328.29; only viruses (+): 1292.55 ± 1240.30; both (-): 634.78 ± 1979.55; both (+): 1427.68 ± 2190.58 pg/mL; p = 0.429; Fig. 4A). Additionally, the sputum hBD-2 level of patients did not show any significant differences at the time of exacerbation according to the pathogen type (only bacteria (+): 2077.38 ± 1329.51; only viruses (+): 1620.73 ± 1297.79; both (-): 2094.72 ± 948.48; both (+): 1990.00 ± 1647.98 pg/mL; p = 0.376; Fig. 4B). However, at the time of exacerbation, the degree of the increase in the sputum hBD-2 level was the highest in both (+) patients and the lowest in the only viruses (+) patients (both (+): 508.76 ± 2026.52; both (-): 1128.92 ± 1041.76; only bacteria (+): 1083.40 ± 1395.53; only viruses (+): 115.58 ± 1611.11 pg/mL; p = 0.07; Fig. 4C).

### Correlation network analysis of cytokines in patients

**Fig. 5:**
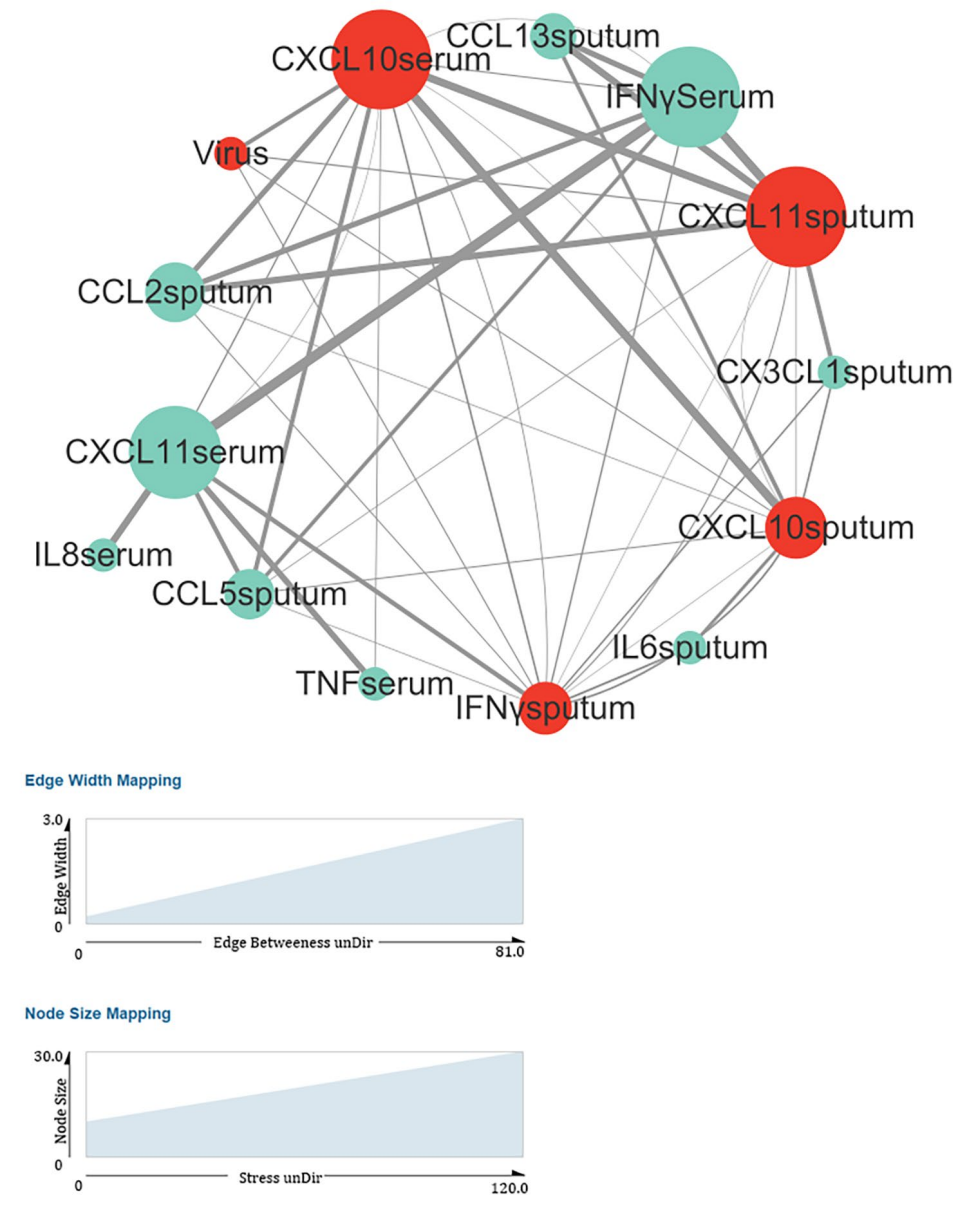
Virus correlation network analysis. At the time of exacerbation, the levels of cytokines, such as CXCL10, CXCL11, and IFN-γ, were significantly associated with virus colonization in patients. Virus-related cytokines are colored red; other cytokines are colored light green (for more details, see the Statistical analysis section)

We performed a correlation network analysis between viruses and cytokine levels (Fig. 5) to evaluate the relationships among various cytokines. We found that most virus-related cytokines were CXCL10, CXCL11, and IFN-γ.

**Fig. 6:**
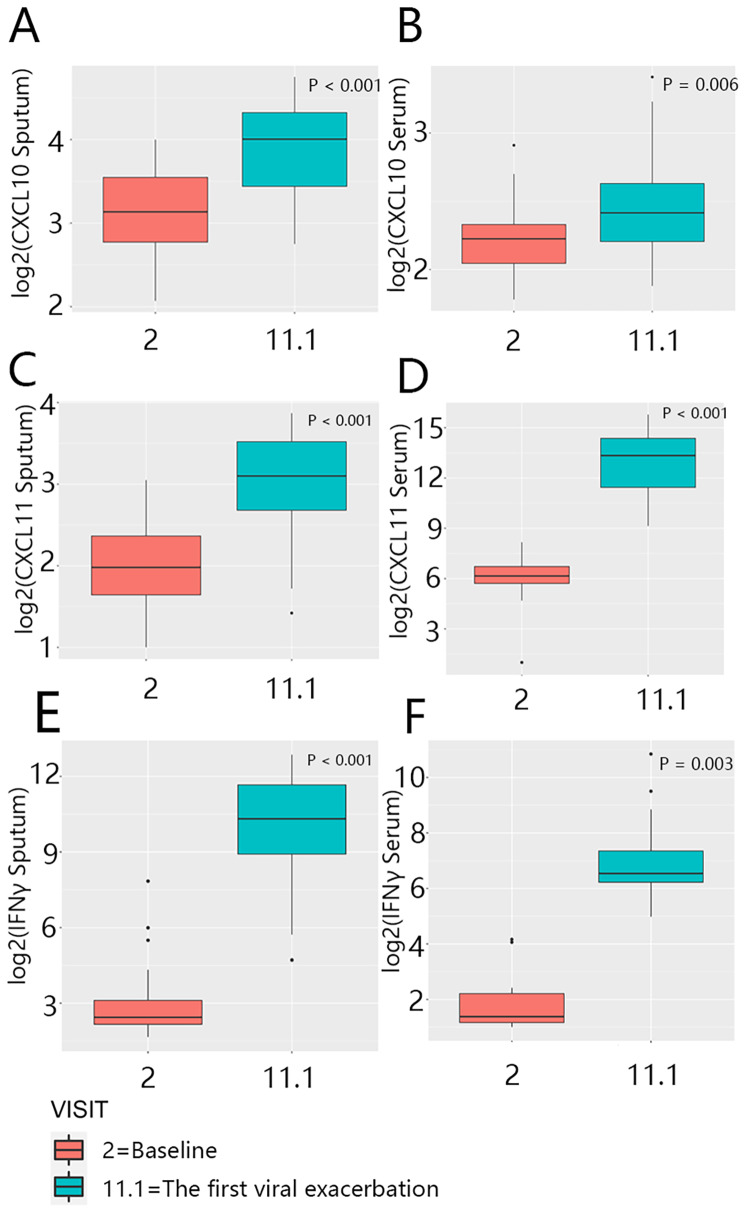
Paired analysis for the comparison of within-patient levels of CXCL10 (A, B), CXCL11 (C, D), and IFN-γ (E, F) at baseline and the first virus (+) exacerbation. Statistical methods: Significance was calculated using the paired Wilcoxon signed-rank test (for more details about Fig. 6, see Additional file 1: Table S7)

In stable disease, the levels of sputum CXCL10, CXCL11, and IFN-γ were not significantly different between patients (CXCL10, serum: p = 0.027; sputum: p = 0.042; CXCL11, serum: p = 0.070; sputum: p = 0.038; IFN-γ, serum: p = 0.137; sputum: p = 0.975; Additional file 2: Figure S1). However, at the time of exacerbations, there were significant differences in the cytokine levels of virus (+) and virus (-) patients (CXCL10, serum: p < 0.001; sputum: p < 0.001; CXCL11, serum: p = 0.048; sputum: p < 0.001; IFN-γ, serum: p = 0.001; sputum: p < 0.001; Additional file 2: Figure S2). Moreover, we compared the cytokine levels in the same patients at baseline and at the time of exacerbation. We found that the cytokine levels were markedly increased (CXCL10, serum: p = 0.006; sputum, p < 0.001; CXCL11, serum: p < 0.001; sputum: p < 0.001; IFN-γ, serum: p = 0.003; sputum: p < 0.001; Fig. 6, A–F).

## Discussion

In this cohort study, we found that the sputum hBD-2 level may predict exacerbations in COPD patients in the next 12 months. Specifically, GOLD classification was negatively correlated with the sputum hBD-2 level, and lower hBD-2 levels usually indicated COPD exacerbations, thereby supporting the notion that host defense mechanisms are important determinants of COPD exacerbations.

High PPMs were found in the sputum samples collected from patients with stable disease (visits 2–4). The bacteria (+) patients with stable disease account for 38% of all patients. These results are consistent with earlier reports that the colonization of PPMs, as determined using baseline sputum samples, was present in the lower airways of 38–73% of patients [7, 8, 27–30]. The percentage of PPM colonization varies between 33% and 50% in samples collected from the lower airways [31–33]. At the time of exacerbations, bacteria (+) patients account for 41% of all patients; however, in an earlier study, this percentage was reported as 50% [12, 34]. Increasing evidence suggests that bacterial infection-induced exacerbations result from changes in the relative abundance of pre-existing bacteria rather than the appearance of new bacterial species or strains [6, 35]. In our cohort study, pathogen colonization at baseline did not predict future COPD exacerbations; however, some previous studies have reported the opposite results [27, 28, 36].

At the time of exacerbations in our study (visits 11.1–11.3), both (+) patients accounted for 23% of all patients (Additional file 1: Table S3); however, in other reports, this percentage was reported as 25%. It seems that these patients are more likely to experience exacerbations and have poor prognoses [13, 30, 37]. During stable disease, both (+) patients accounted for 11% of all patients (Additional file 1: Table S3). It has been suggested that viral colonization may downregulate host defense responses, leading to bacterial proliferation and inflammation. Mallia et al. demonstrated that experimental rhinovirus infection is strongly associated with exacerbations in COPD patients [11]. George et al. found that natural rhinovirus infection in COPD patients was also associated with exacerbations [38]. A possible explanation for these findings could be that induction of the type I IFN response may attenuate the body’s anti-bacterial immune response [39]. The levels of CXCL10, CXCL11, and IFN-γ are mainly associated with viral colonization, especially during exacerbations. It may indicate a strong type I IFN response induced by viral colonization at the time of exacerbations.

Bacterial stimuli can induce hBD-2 through Toll-like receptors (TLRs), and various pro-inflammatory factors, such as TNF-α, IL-1β, and IL-18, can also induce hBD-2 expression. The induction of hBD-2 is regulated by the binding of NF-κB and AP-1 in the hBD-2 promoter [40]. In viral infections, TNF-α activation leads to the induction of hBD-2 via autocrine/paracrine mechanisms [41]. As an anti-microbial peptide binding to bacterial membranes, hBD-2 also contributes to the chemotaxis of CCR6, which expresses immature dendritic cells, and CCR2, which expresses monocytes, macrophages, and neutrophils [1, 4, 42, 43]. Recently, Skronska-Wasek et al. demonstrated that hBD-2 also enhances the phagocytic capacity of macrophages [44].

In a previous study, when COPD patients, healthy smokers, and non-smokers were infected with rhinovirus; only non-smokers had a mild increase in sputum hBD-2 level [11], suggesting that the induction response for hBD-2 was impaired due to rhinovirus infection. Moreover, Milad et al. reported that hBD-2 could significantly attenuate lung inflammation and promote host defense responses induced by bacteria [45].

Some previous studies of the role of hBD-2 have contradictory results. Genetic analysis has revealed an association between the risk of COPD exacerbation and the copy number of hBD-2 [46], but the association has not been found in later studies [47]. In a GWAS study of lung function phenotype, three clusters of hBD genes related to lung function were identified [48]. However, further research is needed.

### Limitations

In this 2-year cohort study, contrary to the study protocol, samples from all patients were not collected, resulting in a relatively small sample size. This may explain the lack of association between the GOLD classification and the exacerbation frequency (Fig. 1E). However, it is also possible that the exacerbation frequency is indeed independent of the hBD-2 levels. Moreover, as a single-center study, our findings cannot be extrapolated to other regions.

## Summary

In conclusion, low hBD-2 levels were associated with an increased risk of exacerbation in the following 12 months. Bacterial colonization may be an important factor that contributes to the increased sputum hBD-2 level at the time of exacerbations. Further studies are needed to explore the relationship between hBD-2 level, pathogen colonization, and exacerbation risk.

## Electronic supplementary material

Below is the link to the electronic supplementary material.

Supplementary Material 1

Supplementary Material 2

## Data Availability

The datasets used and/or analyzed during the current study are available from the corresponding author upon reasonable request.

## Abbreviations

hBD-2: Human beta-defensin-2
COPD: chronic obstructive pulmonary disease
CXCL10: C-X-C motif chemokine 10
CXCL11: C-X-C motif chemokine 11
TLRs: Toll-like receptors
TNF-α: tumor necrosis factor alpha
IL-1: interleukin-1
IL-6: interleukin-6
IL-17A: interleukin-17 A
PN: predicted normal
GINA 2008: The Genetic Information Nondiscrimination Act of 2008
TLC: total lung capacity
FRC: functional residual capacity
IC: inspiratory capacity
RV: residual volume
FEV1: forced expiratory volume in first second
FVC: forced vital capacity
ATS: American Thoracic Society
ERS: European Respiratory Society
GOLD: Global Initiative for Chronic Obstructive Lung Disease
BODE index: a composite index consisting of body mass index, airflow obstruction, dyspnea, and exercise capacity;
ELISA: enzyme-linked immunosorbent assay
IFN-γ: interferon-gamma
PPMs: potential pathogenic microorganisms
NF-κB: nuclear factor kappa-B
AP-1: activator protein 1
CCR6: C-C motif chemokine receptor 6
CCR2: C-C motif chemokine receptor 2
GWAS: genome-wide association analysis
mRNA: messenger ribonucleic acid
PCR: polymerase chain reaction
DL_CO_: diffusion capacity for carbon monoxide.
