# Supplementary material for: Low human beta-defensin-2 levels in the sputum of COPD patients are associated with the risk of exacerbations
Source: BMC Pulm Med. 2023 Mar 31;23:106. doi: 10.1186/s12890-023-02364-0 (PMC10064533; doi:10.1186/s12890-023-02364-0)
Supplement: Supplementary file 1 — Supplementary Material 1 [file 12890_2023_2364_MOESM1_ESM.docx]

**Tables**

Table S1: Viral and bacterial species identified using RT-PCR, including primer and probe sequences.

| **Pathogen** | **Sequences: 5’→3’** |
| --- | --- |
| Influenza A | GAGGTCGAAACGTATGTTCTCTCTATC |
|  | TCTTCAAGTCTCTGCGCGATT |
|  | FAM-CATCAGGCCCCCTCAAAGCCG-BHQ1 |
| Influenza B | CCCTGCTTGCTCGTAGTATGG |
|  | GCTTATGGGAAGCACCACTTTG |
|  | FAM-CGTTGTTAGGCCCTCTGTGGCGA-BHQ |
| Parainfluenza-1 | TGTAGGAAGTGGGATAAAGATTGAAA |
|  | GTGTCGCCTTGGAGCGGAGTT |
|  | FAM-TACACTCGTTTTCCTAGGGTACGGTGGCTT-BHQ |
| Parainfluenza -2 | AAGTGACAACCAGCAGAGATTCAA |
|  | TGTTGGAACTCTTCACTGCTCATAC |
|  | FAM-CCCCCCATACCGCAAAGAACATCA-BHQ |
| Parainfluenza -3 | CAACCATATGCTGCGCTATACC |
|  | CAAGACCTCCATATCCGAGAAATATT |
|  | FAM-ATCTGTTGGACCAGGGATATACTACAAAGGCAA-BHQ |
| Parainfluenza -4 | CCGGCAAGCTGTATCATCAT |
|  | CCGACTTGCCGTATCATCAT |
|  | TGTAAGGAAAGCAGAGATAATAATGC |
|  | FAM- TGCCAAATCGGCAATTAAACAGGG-BHQ |
| RSV | CAGTAGAGTTGAAGGGATTTTTGCA |
|  | CCCCCCACCGTAACATCAC |
|  | FAM-ATTGTTTATGAATGCCTATGGTGCAGGGC-BHQ |
|  | TAGTAGAGTTGAAGGAATCTTTGCA |
|  | TCCCCCATCTTAGCATTAC |
| Enterovirus | GAAGAGTCTATTGAGCTA(A/G)TT(G/A)GTAGTCCT |
|  | GAAACACGGACACCCAAAGTAGT |
|  | FAM-CGGCCCCTGAATGCGGCTAATC-BHQ1 |
| Adenovirus | ATCGATGATGCCCCAATGG |
|  | GGACTCAGGTACTCCGAAGCA |
|  | FAM-CAT ACA TGC ACA TCG CCG GAC AGG-BHQ |
| Rhinovirus 1 | CTTTGAGTCCTCCGGCCC |
|  | CCCGCAATTGCTCATTACGAC |
|  | FAM-TGAATGTGGCTAACCTTAACCCTGCAGC-BHQ |
| Rhinovirus 2 | CCCCACTGGCGACAGTGT |
|  | GGGGCTCTTCACACCTTGTC |
|  | FAM-CTAGCCTGCGTGGCTGCCTGC-BHQ |
| Rhinovirus 3 | CCACTAGTTTGGTCGATGAGGCT |
|  | TGGCCGCCACGCAGG |
|  | FAM-GAATTCCCCACGGGCGACCGTGTC-BHQ |
| Rhinovirus 4 | TGTGAAGAGCCCCGTGTGCT |
|  | TGCAGGGTTAAGGTTAGCCACAT |
|  | FAM-CAGGGGCCGGAGGAC-MGB |
| Human Metapneumovirus1 | TGCTATATTAAAAGAGTCCCAATACACAAT |
|  | CTCTTGCTGCAATGATGAAGGT |
|  | FAM-AGAGATGTAGGCACCACAAC-MGB |
| Human Metapneumovirus 2 | CCAAAGGCAGGACTGTTATCG |
|  | CCTGAAGCATTACCAAGAACAACAC |
|  | FAM-TGGCCAATTGCCCCAATTTTGC-BHQ |
| Human Metapneumovirus 3 | GCCCAAAAGCTGGACTGTTATC |
|  | GAGGCATTTCCGAGAACAACAC |
|  | FAM-CTAGCCAACTGTCCCAACTTTGCA-BHQ |
| Coronavirus 229E | CAGGTGTTGTGGCCAATGG |
|  | GCTGTTGACGGCACAAGCT |
|  | FAM-TTAAAGCTAAAGGCTATCCACAATTTGC-BHQ |
| Coronavirus OC43 | ATACCCCGGCTGACATTGTC |
|  | AGGCGGAAACCTAGTCGGAAT |
|  | FAM-TCGGGACCCAAGTAGCGATGAGGC-BHQ |
| Mycoplasma pneumoniae (MP) | GGG AGG CAG CAG TAG GGA AT |
|  | GCG GCA TTG CTC CAT CA |
|  | FAM-TTT CAC AAT GAG CGA AAG-MGB |
| Chlamydia pneumoniae (CP) | TGA AGT CGG AAT TGC TAG TAA TGG |
|  | AAG GCC CGA GAA CGT ATT CAC |
|  | FAM-TGT CAG CCA TAA CGC-MGB |
| Legionella pneumophila (LP) | TCCGGAAGCAATGGCTAAAG |
|  | TGCTGTTCGGTTAAAGCCAAT |
|  | FAM-CATGCAAGACGCTATGAGTGGCGC-BHQ |

Table S2: Bacterial species identified using sputum culture.

| **Pathogen** |
| --- |
| *Pseudomonas aeruginosa* |
| *Escherichia coli* |
| *Acinetobacter baumannii* |
| *Klebsiella pneumoniae* |
| *Serratieae* |
| *Flavobacterium* |
| *Stenotrophomonas Maltophilia* |
| *Enterobacter cloacae* |
| *Enterbacter aerogenes* |
| *Branhamaceae catarrhalis* |
| *Proteus mirabilis* |
| *Staphylococcus epidermidis* |
| *Staphylococcus aureus* |
| *Steptococcus mitis* |
| *Enterococcus* |
| *Haemophilus influenzae* |
| *Streptococcus pneumoniae* |
| *Legionella* |
| *Moraxella catarrhalis* |

Table S3: Number and proportion (Bact +, Bact -, Vir +, Vir -) of participants at all visits (2–4 and 11.1–11.3), different GOLD classification classes (GOLD 0, 1, 2, and 3–4), and number of controls. Bact +, bacteria positive; Bact -, bacteria negative; Vir +, viruses positive; and Vir -, viruses negative. Visit 2: baseline; visit 3: 12 months; and visit 4: 24 months. Visits 11.1–11.3: exacerbation.

|  | **Bact+ N(%)** | **Bact-**  **N** | **Vir+**  **N(%)** | **Vir-**  **N** |
| --- | --- | --- | --- | --- |
| Visit 2 | 140(57) | 105 | 50(20) | 203 |
| Visit 3 | 65(30) | 150 | 79(36) | 138 |
| Visit 4 | 49(23) | 161 | 81(38) | 132 |
| Visit 2-4 | 254(38) | 416 | 210(31) | 473 |
| Visit 11.1-11.3 | 64(41) | 92 | 82(52) | 76 |
| GOLD 0 | 73(41) | 102 | 55(31) | 124 |
| GOLD 1 | 50(33) | 101 | 53(35) | 99 |
| GOLD 2 | 70(39) | 108 | 71（39） | 110 |
| GOLD 3-4 | 72(37) | 122 | 69(35) | 130 |
| GOLD 0-4 | 265(38) | 433 | 248(35) | 463 |
| Control | 53(41) | 75 | 46(35) | 84 |

Positivity was defined as testing positive for any pathogens shown in Table S2, on either a nasal swab or sputum. Samples from all visits were included.

Table S4: Number and proportion (Both -, Both +, Only Bact +, Only Vir +) of participants at all visits (2–4 and 11.1–11.3), different GOLD classification classes (GOLD 0, 1, 2, and 3–4), and number of controls. Both -, both bacteria and viruses negative; Both +; both bacteria and viruses positive; Only Bact +, only bacteria positive; and Only Vir +, only viruses positive.

|  | **Both -**  **N (%)** | **Only Bact +**  **N (%)** | **Only Vir +**  **N (%）** | **Both+**  **N (%)** |
| --- | --- | --- | --- | --- |
| Visit 2 | 53 | 73 | 15 | 16 |
| Visit 3 | 97 | 39 | 53 | 26 |
| Visit 4 | 101 | 28 | 60 | 21 |
| Visit 2-4 | 252(43) | 140(24) | 128(22) | 63(11) |
| Visit 11.1 | 13 | 7 | 16 | 11 |
| Visit 11.2 | 5 | 7 | 5 | 4 |
| Visit 11.3 | 0 | 3 | 2 | 2 |
| Visit 11.1-11.3 | 18(24) | 17(23) | 23(30) | 17(23) |
| GOLD O | 66(38) | 54(31) | 36(20) | 19(11) |
| GOLD 1 | 63(42) | 34(23) | 37(24) | 16(11) |
| GOLD 2 | 62(35) | 44(25) | 45(25) | 26(15) |
| GOLD 3-4 | 81(42) | 43(22) | 40(21) | 29(15) |
| GOLD 0-4 | 319(39) | 210(26) | 186(22) | 108(13) |
| Control | 47(37) | 35(27) | 28(22) | 18(14) |

Positivity was defined as testing positive for any pathogens shown in Table S2, on either nasal swabs or sputum. Samples from all visits were included.

Table S5: More details about Figures 1 and 3 (mean ± SD).

| Figures | Groups | hBD-2_ sputum |  | hBD-2_ serum |
| --- | --- | --- | --- | --- |
| Figure 1A | control | 2152.5 ± 1251.6 | Fig 1B | 3899.1±2561.3 |
|  | GOLD 0-4 | 1716.9 ± 1248.6 |  | 5204.4±3704.7 |
| Figure 1C | without any exacerbation | 2103.7±1294.2 |  |  |
|  | exacerbation more than once | 1130.9±858.4 |  |  |
| Figure 1D | control | 2152.5±1251.6 |  |  |
|  | patients without any exacerbation | 2084.9±1317.6 |  |  |
| Figure 3C | GOLD 0 | 1826.30±1353.02 |  |  |
|  | GOLD 1 | 2165.52±1303.80 |  |  |
|  | GOLD 2 | 1518.09±999.67 |  |  |
|  | GOLD 3-4 | 1355.53±1190.34 |  |  |
|  | control | 2216.34±1298.22 |  |  |

Table S6: More details about Figure 4. Relationship between sputum hBD-2 levels and the pathogen colonization status (Only Bact+, Only Vir+, Both+, Both-) at the time of exacerbations (mean ± SD).

| **colonization status** | **Fig. 4A** | **Fig. 4B** | **Fig. 4C** |
| --- | --- | --- | --- |
| Both + | 1427.68 ± 2190.58 | 1990.00 ± 1647.98 | 508.76±2026.52 |
| Both - | 634.78 ± 1979.55 | 2094.72 ± 948.48 | 1128.92±1041.76 |
| Only Bact + | 371.48±1328.29 | 2077.38±1329.51 | 1083.40±1395.53 |
| Only Vir + | 1292.55 ± 1240.30 | 1620.73±1297.79 | 115.58 ±1611.11 |
| P value | P=0.429 | p=0.376 | p=0.07 |

Table S7: More details about Figures S1, S2 and 6. At visits 2–4 (Figure S1) and 11.1–11.3 (Figure S2), CXCL10, CXCL11, and IFN-γ levels in patients subdivided by viral colonization status (Vir + or Vir -) are shown. Paired analysis comparing within-patient levels of CXCL10 (A, B), CXCL11 (C, D), and IFN-γ (E, F) at baseline and the first virus (+) exacerbation (Figure 6).

| **Figures** | **Visits** | **Viruses** | **log2(CXCL10_sputum)** | **log2(CXCL10_serum)** | **log2(CXCL11_sputum)** | **log2(CXCL11_serum)** | **log2(IFN_sputum)** | **log2(IFN_serum)** |
| --- | --- | --- | --- | --- | --- | --- | --- | --- |
| Fig.S1 | 2 | + | 9.44±1.94 | 7.68±0.94 | 5.98±1.77 | 6.19±0.80 | 2.35±0.54 | 2.26±1.34 |
|  | 2 | - | 10.09±1.74 | 7.26±0.56 | 6.29±1.90 | 6.12±1.05 | 3.15±1.65 | 1.61±0.91 |
|  | 3 | + | 9.93±1.18 | 7.45±0.81 | 5.85±1.38 | 6.48±0.89 | 2.54±0.79 | 2.19±1.55 |
|  | 3 | - | 9.73±1.27 | 7.38±0.77 | 5.39±1.27 | 6.15±0.71 | 3.19±1.39 | 1.45±0.66 |
|  | 4 | + | 10.80±1.98 | 7.48±0.67 | 6.80±2.47 | 6.42±0.70 | 3.40±2.02 | 1.82±0.76 |
|  | 4 | - | 9.72±1.33 | 7.17±0.52 | 5.62±1.40 | 6.13±1.38 | 2.13±0.43 | 1.27±0.41 |
| Fig.S2 | 11.1 | + | 13.00±1.82 | 8.11±1.14 | 9.88±2.27 | 6.80±1.13 | 5.13±1.76 | 2.32±1.11 |
|  | 11.1 | - | 10.01±1.68 | 7.21±0.69 | 6.13±1.77 | 6.47±0.98 | 2.81±0.75 | 1.39±0.45 |
|  | 11.2 | + | 12.10±2.88 | 8.11±1.30 | 8.96±2.94 | 6.49±2.09 | 4.85±1.30 | 2.34±0.79 |
|  | 11.2 | - | 10.39±1.91 | 7.43±0.98 | 6.85±1.96 | 6.56±1.02 | 3.71±0.92 | 2.07±1.71 |
|  | 11.3 | + | 11.32±2.56 | 8.55±1.62 | 7.43±2.49 | 7.27±1.95 | 5.27±2.71 | 2.42±1.12 |
|  | 11.3 | - | 9.67±1.24 | 6.88±0.43 | 5.91±0.65 | 6.03±0.55 | 2.41 | 0.69 |
| Fig.6 | 2 | / | 9.82±1.87 | 7.16±0.56 | 6.31±2.03 | 5.92±1.18 | 2.82±1.35 | 0.68±0.95 |
|  | 11.1 | + | 13.00±1.86 | 8.11±1.14 | 9.86± 2.40 | 6.80±1.13 | 4.03±1.65 | 2.11±1.76 |
