# Supplementary material for: Low human beta-defensin-2 levels in the sputum of COPD patients are associated with the risk of exacerbations
Source: BMC Pulm Med. 2023 Mar 31;23:106. doi: 10.1186/s12890-023-02364-0 (PMC10064533; doi:10.1186/s12890-023-02364-0)

**Figures**

Figure S1: At visits 2–4, the CXCL10, CXCL11, and IFN-γ levels were compared in patients according to the viral colonization status (Vir + or Vir -). CXCL10 (A–B), CXCL11 (C–D), and IFN-γ (E, F). 2 = visit 2 (baseline); 3 = visit 3 (1-year assessment); and 4 = visit 4 (2-year assessment). Statistical methods: Wilcoxon signed-rank test was used to calculate significance (for more details about Figure S1, see Additional file 1: Table S7).


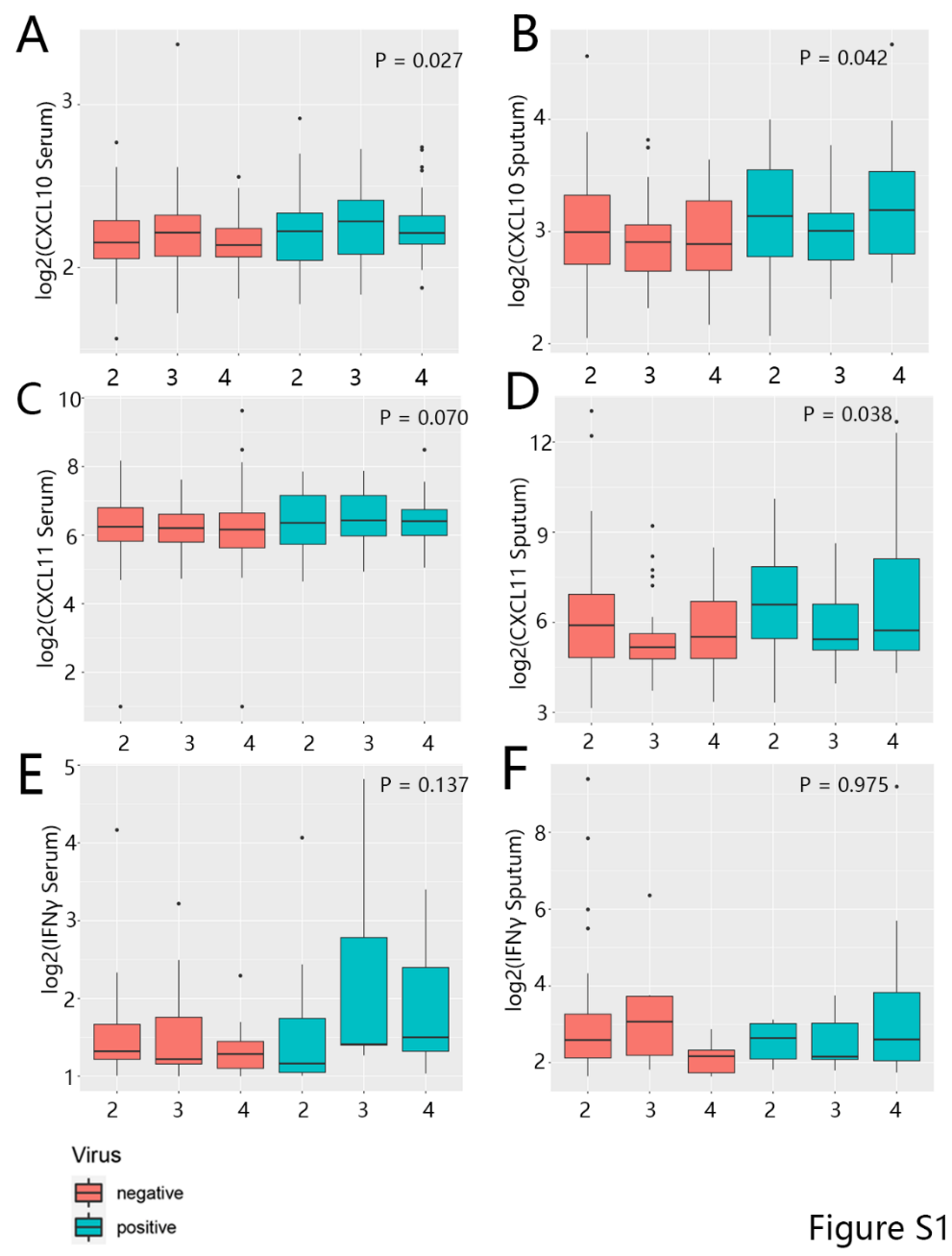


Figure S2: At the time of exacerbations (visits 11.1–11.3), the CXCL10, CXCL11, and IFN-γ levels were compared in patients according to the viral colonization status (Vir + or Vir -). CXCL10 (A–B), CXCL11 (C–D), and IFN-γ (E, F). 11.1 = first exacerbation; 11.2 = second exacerbation; and 11.3 = third exacerbation. Statistical methods: Wilcoxon signed-rank test was used to calculate significance (for more details about Figure S2, see Additional file 1: Table S7).


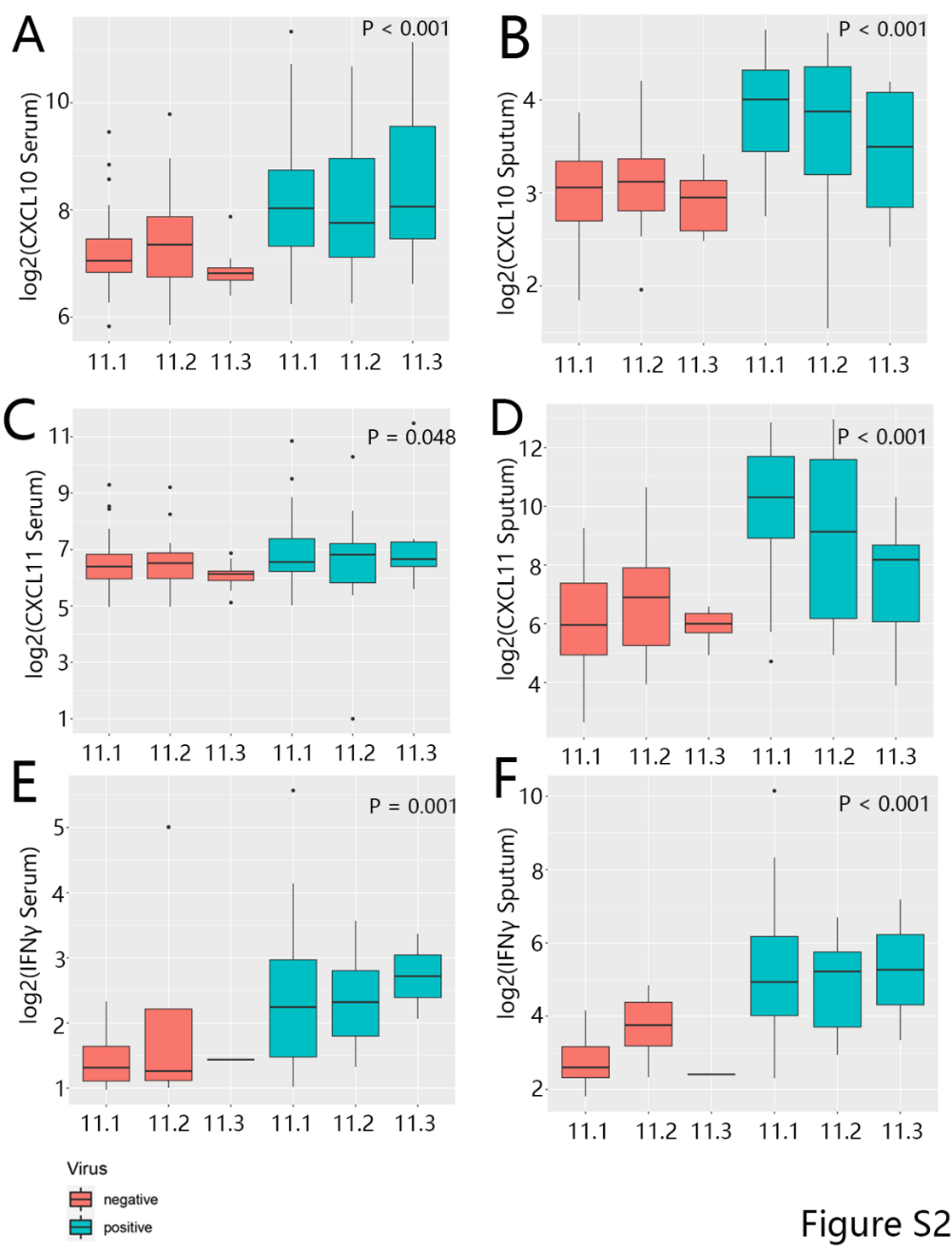

Supplement: Supplementary file 2 — Supplementary Material 2 [file 12890_2023_2364_MOESM2_ESM.docx]
